# Supplementary material for: Differential Relationships Between Brain Structure and Dual Task Walking in Young and Older Adults
Source: Front Aging Neurosci. 2022 Mar 11;14:809281. doi: 10.3389/fnagi.2022.809281 (PMC8963788; doi:10.3389/fnagi.2022.809281)
Supplement: Supplementary file 1 [file Data_Sheet_1.PDF]

Supplementary Material

1 PARTICIPANT INCLUSION AND EXCLUSION INFORMATION

1.1 Participant Screening

We screened all subjects for MRI eligibility and, as part of the larger study, transcranial magnetic stimulation (TMS) eligibility. We excluded those with any MRI or TMS contraindications (e.g., implanted metal, claustrophobia, or pregnancy). We also excluded individuals with: history of any neurologic condition (e.g., stroke, Parkinson's disease, seizures, or a concussion in the last six months); a current psychiatric condition (e.g., active depression or bipolar disorder); self-reported smokers; those who self-reported consuming more than two alcoholic drinks per day on average; and those with history of treatment for alcoholism. All participants were right-handed and self-reported their ability to walk unassisted for at least 10 minutes and to stand for at least 30 seconds with their eyes closed.

Prior to enrollment, we screened participants for suspected cognitive impairment over the phone using the Telephone Interview for Cognitive Status (TICS; de Jager et al., 2003). We excluded those who scored $<$ 21 of 39 points; this is equivalent to scoring $<$ 25 points on the Mini-Mental State Exam (MMSE) and indicates probable cognitive impairment (de Jager et al., 2003). At the first testing session, we re-screened participants for cognitive impairment using the Montreal Cognitive Assessment (MoCA; Nasreddine et al., 2005). We added one point to the scores of participants with $\leq$ 12 years of education (Nasreddine et al., 2005). We did not enroll those who scored $<$ 23 of 30 points (Carson et al., 2018).

1.2 MRI Scan Exclusions

Two older adults were excluded from analyses of the T_1-weighted images. One of these older adults did not fit within the 64-channel coil, so a 20-channel coil was used instead; due to low image quality, we excluded their data from further analysis. The other older adult T_1-weighted scan was excluded due to an incidental brain tumor finding. Thus, n = 23 older adults for all analyses involving the T_1-weighted images. Due to time constraints, a diffusion MRI was not collected for one young and two older adults; thus, n = 36 young and n = 21 older adults for all diffusion MRI analyses.

2 METHODS FOR PROCESSING OF T_1-WEIGHTED IMAGES

Here we provide further details regarding the preprocessing of the T_1-weighted images.

2.1 Gray Matter Volume

We processed the T_1-weighted scans using the Computational Anatomy Toolbox toolbox (version r1725; Gaser et al., 2016; Gaser and Kurth, 2017) in MATLAB (R2019b). We implemented default CAT12 preprocessing steps, including the new adaptive probability region-growing skull stripping method. Briefly, the CAT12 pipeline includes segmentation into gray matter, white matter, and cerebrospinal fluid, followed by spatial normalization from subject space to standard space using high-dimensional Dartel registration and modulation. After CAT12 preprocessing was complete, we visually examined data quality by displaying each modulated, normalized gray matter segment and checking alignment between subjects and with the standard space template.

We did not remove any scans as a result of visual inspection. All scans passed acceptable CAT12 quantitative quality control thresholds (i.e., resolution, noise, bias, and image quality > 80). Finally, we used the CAT12 *Check Sample Homogeneity* function to evaluate correlations between all gray matter segments. Gray matter segments for each participant were within two standard deviations of the group mean, indicating that the sample contained no outliers. To increase signal-to-noise ratio, we smoothed the modulated, normalized gray mattersegments using Statistical Parametric Mapping 12 (SPM12, v7771; Ashburner et al., 2014) with an 8 mm full width at half maximum kernel. We entered these preprocessed gray matter volume maps into the group-level voxelwise statistical models. We used CAT12 to calculate total intracranial volume for each participant for later use as a covariate in these group-level statistical analyses.

2.2 Cortical Surface Metrics

The CAT12 pipeline also extracts surface-based morphometry metrics (Dahnke et al., 2013; Yotter et al., 2011a). To calculate surface metrics, CAT12 uses a projection-based thickness algorithm that handles partial volume information, sulcal blurring, and sulcal asymmetries without explicit sulcus reconstruction (Dahnke et al., 2013; Yotter et al., 2011a). We used CAT12 to extract four surface metrics: 1) cortical thickness: the thickness of the cortical gray matter between the outer surface (i.e., the gray matter-cerebrospinal fluid boundary) and the inner surface (i.e., the gray matter-white matter boundary) (Dahnke et al., 2013); 2) cortical complexity: fractal dimension, a metric of folding complexity of the cortex (Yotter et al., 2011b); 3) sulcal depth: the Euclidean distance between the central surface and its convex hull (Yun et al., 2013); and 4) gyrification index: a metric based on the absolute mean curvature, which quantifies the amount of cortex buried within the sulcal folds as opposed to the amount of cortex on the "outer" visible surface (Luders et al., 2006). Prior to further analysis, we visually checked all cortical surface data using CAT12's *Display Surfaces* tool and then resampled and smoothed the surfaces at 15 mm for cortical thickness and 20 mm for the three other metrics (Gaser and Kurth, 2017). We entered these resampled and smoothed surface files into the group-level voxelwise statistical models.

2.3 Cerebellar Volume

To improve the normalization of the cerebellum (Diedrichsen, 2006; Diedrichsen et al., 2009), similar to our past work (Hupfeld et al., 2021; Salazar et al., 2020, 2021), we applied specialized preprocessing steps to the cerebellum to produce cerebellar volume maps. First, we entered each participant's whole-brain T_1-weighted image into the CEREbellum Segmentation (CERES) pipeline (Romero et al., 2017). CERES uses a patch-based segmentation approach to segment the cerebellum from the cortex; this automated method has been demonstrated to perform better than either semi-automatic or manual cerebellar segmentation (Romero et al., 2017). We visually inspected the resulting segmentations, created a binary mask from each participant's CERES cerebellar segmentation, and used this mask to extract their cerebellum from their whole-brain T_1-weighted image. We then used rigid, affine, and Symmetric Normalization (SyN) transformation procedures within the Advanced Normalization Tools package (ANTs; v1.9.17; Avants et al., 2010, 2011) to warp (in a single step) each participant's extracted subject space cerebellum to a 1 mm cerebellar template in standard space, the Spatially Unbiased Infratentorial Template (SUIT) template (Diedrichsen, 2006; Diedrichsen et al., 2009). The SUIT template was selected because it offers greater detail of internal cerebellar structures compared to whole brain templates, which improves cerebellar normalization (Diedrichsen, 2006; Diedrichsen et al., 2009). For this warping

we used a version of the SUIT template with the brainstem removed, as the CERES cerebellar segmentation does not include the brainstem.

The flowfields that were applied to warp these cerebellar segments to SUIT space were additionally used to calculate the Jacobian determinant image, using ANTs' *CreateJacobianDeterminantImage.sh* function; the Jacobian determinant encodes local shrinkage and expansion for each voxel between subject space and the target image (i.e., here, the standard space template). We multiplied each normalized cerebellar segment by its corresponding Jacobian determinant to produce modulated cerebellar images in standard space for each participant. Modulation preserves the volumes present in the original untransformed (subject space) image. Lastly, to increase signal-to-noise ratio, we smoothed the modulated, normalized cerebellar images using a kernel of 2 mm full width at half maximum and entered the resulting cerebellar volume maps into the group-level voxelwise statistical models. Of note, we examined cerebellar total volumes in our statistical analyses instead of segmenting the cerebellum by tissue type, in order to avoid any inaccuracy due to low contrast differences between cerebellar gray and white matter.

3 METHODS FOR PROCESSING OF DIFFUSION-WEIGHTED IMAGES

Here we provide more specific details regarding the preprocessing of the diffusion-weighted images.

3.1 Visual Inspection and Signal Drift Correction

We first visually inspected raw scans for artifacts and excessive head movement. We then corrected the diffusion-weighted images for signal drift (Vos et al., 2017) using the ExploreDTI graphical toolbox (University Medical Center Utrecht, Netherlands, Version 4.8.6; `www.exploredti.com`; Leemans et al., 2009) in MATLAB (R2019b).

3.2 Topup

We used the FMRIB Software Library (FSL)'s processing tool `topup` to estimate the susceptibility-induced off-resonance field (Andersson et al., 2003). We entered a pair of b_0 images collected with reversed phase-encode blips (i.e., the first volume of the diffusion-weighted sequence with Anterior to Posterior encoding and one b_0 volume from the Posterior to Anterior sequence collected immediately before the diffusion-weighted sequence). This procedure yielded a single corrected field map for use in eddy current correction.

3.3 Eddy

We used FSL's `eddy_cuda` to simultaneously correct the data for eddy current-induced distortions and both inter- and intra-volume head movement (Andersson and Sotiropoulos, 2016). We entered the topup-calculated field map with the `--topup` flag. We used the `--repol` flag to remove slices classified as outliers (i.e., where signal has been lost due to subject movement during the diffusion encoding) and replace these slices with non-parametric predictions by the Gaussian Process (Andersson et al., 2016). We set `--slm=1`, which specifies the mathematical form for how the diffusion gradients cause eddy currents; this setting is recommended when data are sampled on the half sphere, which was the case here. We used the `--estimate-movement-by-susceptibility` flag, which provides additional corrections to account for the effects of head movement on the diffusion signal (Andersson et al., 2018). We set `--mporder=17` to perform slice-to-volume movement correction (Andersson et al., 2017),

which is a novel (but computationally expensive) method that corrects for within-volume head movement. We set the temporal order of movement at 17, as FSL documentation recommends using the number of excitations divided by 4 (i.e., # of slices / 4, so 69/4 $\approx$ 17). During `eddy_cuda`, rotations applied to each volume during motion correction were also applied to the corresponding *b* vectors. We then plotted each subject's volume-wise root mean square displacement provided by `eddy_cuda`. We considered a volume to be an outlier if its displacement was greater than 1 mm relative to the previous volume. Eight young and four older adults had one or more outlier volumes removed (young adults: 2-8 volumes removed per subject; older adults: 1-3 volumes removed per subject). Outlier volumes were removed from the eddy corrected image, as well as from the *b* value and rotated *b* vector matrices.

3.4 Free-water (FW) Correction

We implemented a custom FW algorithm (Pasternak et al., 2009) using MATLAB (R2019b). This algorithm estimates FW volume by fitting a bitensor model at each voxel of the preprocessed DWI image (Pasternak et al., 2009). The bitensor model consists of: 1) a tissue compartment, i.e., the diffusion indices (including FA, RD, and AD) of water molecules within white matter tissue; and 2) a FW compartment, reflecting the proportion of water molecules with unrestricted diffusion. FW fractional volumes range from 0 to 1; a fraction of 1 indicates that a voxel is filled with freely diffusing water molecules (e.g., as in the ventricles). The outputs of interest from this algorithm include a whole-brain FW map and FW-corrected whole-brain maps of white matter indices, denoted by subscript "t" to indicate that these metrics are based on the tissue compartment (FAt, RDt, and ADt).

3.5 Tract-Based Spatial Statistics (TBSS)

We applied FSL's tract-based spatial statistics (TBSS) processing steps to prepare the data for voxelwise analyses across participants (Smith et al., 2006). Benefits of TBSS include avoiding problems associated with suboptimal image registration between participants and eliminating the need for spatial smoothing. TBSS uses a carefully tuned nonlinear registration and projection onto an alignment-invariant tract representation (i.e., the mean FA skeleton); this process improves the sensitivity, objectivity, and interpretability of analyses of multi-subject diffusion studies.

First, we used `tbss_1_preproc` to erode the FA images slightly and zero the end slices (to remove likely outliers from the diffusion tensor fitting). Next, we used `tbss_2_reg` to calculate the warps to bring each subject's FA data to a common space (i.e., the FMRIB58_FA 1 mm isotropic template) using the nonlinear registration tool FNIRT (Andersson et al., 2007b,a), which employs a b-spline representation of the registration warp field (Rueckert et al., 1999). We then used `tbss_3_postreg` to apply the warps calculated in step two, to calculate a mean FA image, and to thin this mean image to create a mean FA skeleton. This mean FA skeleton represented the centers of all tracts common to the whole group. Finally, we used `tbss_4_prestats` with a threshold of 0.2 to project each participant's aligned FA data onto the group mean skeleton.

Lastly, we applied FSL's `TBSS_non_FA` script to the additional whole-brain maps (i.e., the FW, FAt, RDt, and ADt maps). This applied the original nonlinear registration to these maps and projected the data onto the original mean FA skeleton (using the original FA data to find the projection vectors). Ultimately, these TBSS procedures resulted in skeletonized FW, FAt, ADt, and RDt maps in standard space for each participant. These were the maps that we entered in the group-level voxelwise statistical models.

3.6 Image Processing for Region of Interest Analyses

CAT12 automatically calculates the inverse warp, from standard space to subject space, for several volume-based atlases. We isolated multiple regions of interest (ROIs) from these atlases in subject space: the lateral ventricles and pre- and postcentral gyri from the Neuromorphometrics (`http://Neuromorphometrics.com`) volume-based atlas, and the thalamus, striatum, and globus pallidus from the CoBra Subcortical atlas (Tullo et al., 2018; Fig. S1). We visually inspected each ROI mask overlaid onto each participant's T_1-weighted image in ITK-SNAP and hand corrected the ROI mask if needed (i.e., if any voxels were not over the pre-specified region) (Yushkevich et al., 2006). Using *fslstats*, we extracted the number of voxels in each ROI mask in subject space and calculated the mean image intensity within the ROI in the subject space cerebrospinal fluid (lateral ventricles) or gray matter segment (for all of the other ROIs). We then calculated ROI volume in mL as: (number of voxels in the ROI mask)*(mean intensity of the tissue segment within the ROI mask)*(volume/voxel). In subsequent statistical analyses, we used the average of the left and right side structures for each ROI, and we entered these ROI volumes as a percentage of total intracranial volume (to account for differences in head size).

3.7 FW ROIs

We also extracted FW values from the diffusion MRI maps for the same ROIs for which we calculated gray matter volume. We rigidly registered the subject space T_1-weighted image to the subject space FW image. (We used a rigid registration in this case because we previously used *topup* to resolve distortions during diffusion-weighted preprocessing). We then used ANTs to apply the inverse of that transformation to the subject T_1-space atlases described above. This resulted in volumetric atlases for each participant in their native diffusion space. We then isolated masks for the same ROIs described above from these atlases and visually inspected each ROI mask overlaid onto each participant's FW map in ITK-SNAP. Finally, we used *fslstats* to extract mean image intensity in the FW map within each ROI mask. Here we used mean intensity as our outcome metric (rather than volume in mL as above) to estimate the fractional volume of FW within the ROI and obtain a metric more representative of microstructural FW, rather than the size of the ROI which represents macrostructural atrophy. We calculated the average mean intensity for the left and right side for each structure and used this average value in subsequent statistical analyses.

3.8 Hippocampal ROIs

We implemented the Automatic Segmentation of Hippocampal Subfields (ASHS)-T1 (Yushkevich et al., 2015) pipeline within ITK-SNAP (Yushkevich et al., 2015) to segment and extract the volume in mL of three hippocampal structures: anterior hippocampus, posterior hippocampus, and parahippocampal cortex. The ASHS pipeline uses a multi-atlas segmentation framework and super-resolution approach; this outperforms alternative T_1 hippocampal segmentation pipelines by reducing misclassification of meninges as gray matter (Yushkevich et al., 2015). Though this pipeline is currently validated for use on only older adults (defined as those 55+ years old; Yushkevich et al., 2015), for completeness, here we also implemented the pipeline on my younger adult participants. For statistical analyses, we used the average of the left and right side structures, and we entered these volumes as a percentage of total intracranial volume (to account for differences in head size).

4 SUPPLEMENTARY FIGURES AND TABLES

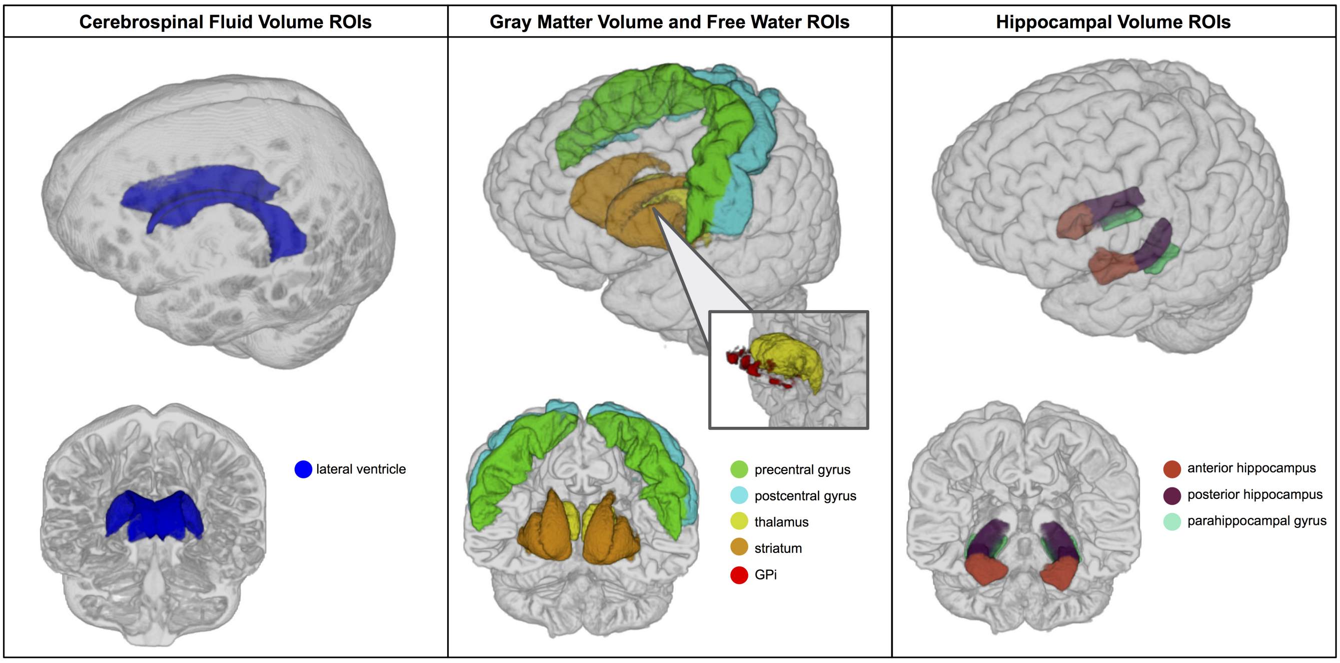

Figure S1. Structural ROIs. Here we depict ROI masks overlaid onto subject space cerebrospinal fluid (left) and gray matter (middle, right) segments for an exemplar young adult participant. In every case, we used the average of the left and right side ROI in our statistical analyses. Left. Lateral ventricle ROI masks. Middle. Five ROIs for which we extracted both gray matter volume and FW. ROIs are shown over the gray matter segment obtained from the T_1-weighted image. We do not depict here the subject space FW image from which the FW values were obtained. See Fig. S4 for illustrations of these ROIs overlaid onto the FW image. Right. Three hippocampal ROIs.

.

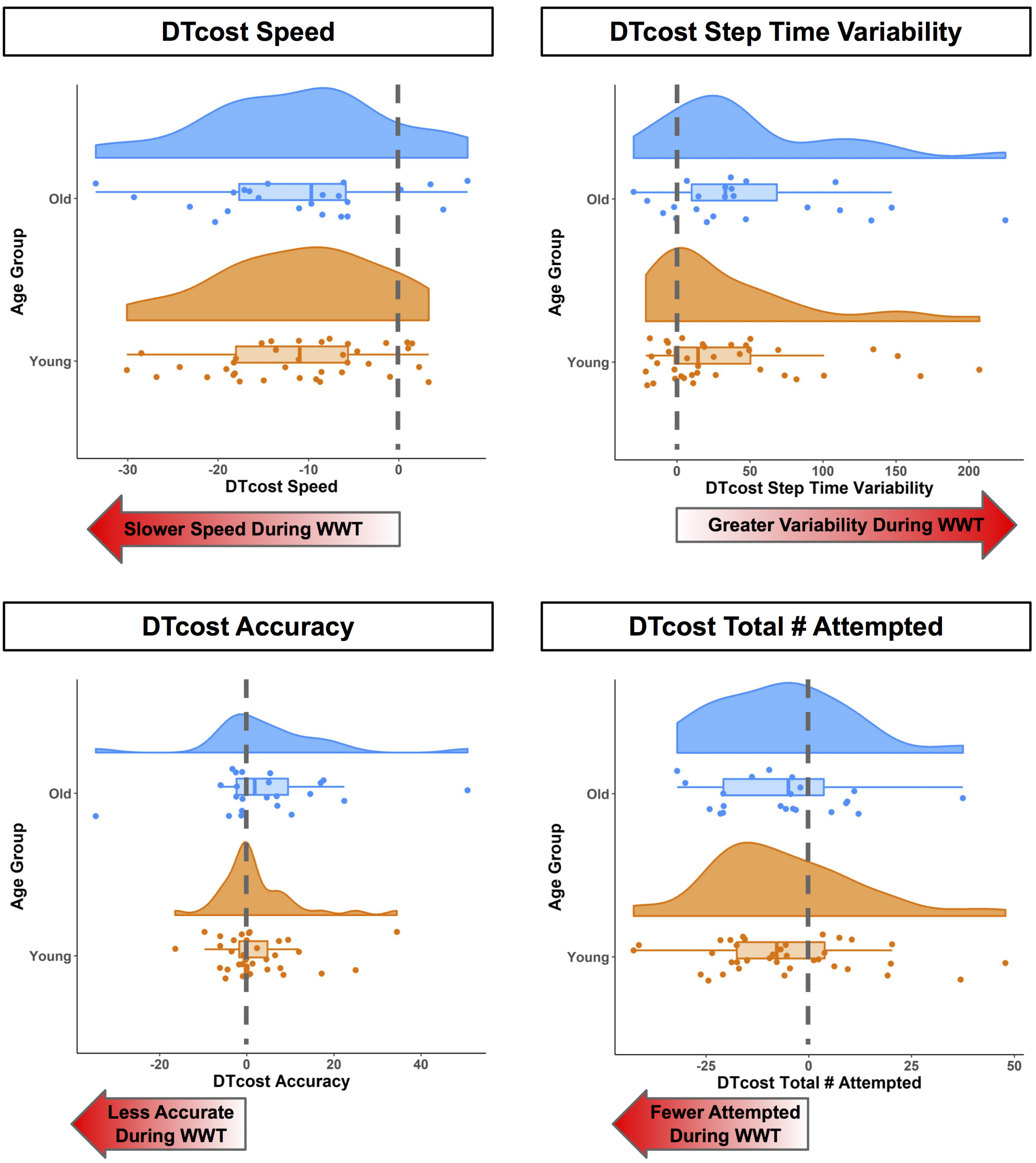

Figure S2. No age differences in the DTcost of gait and subtraction performance. The DTcost of gait and subtraction performance metrics is depicted for older (blue) and young (orange) adults. The red arrows indicate the direction of poorer performance during the dual compared to the single task conditions. There were no statistically significant age group differences in the DTcost of gait or serial subtraction performance. Gait speed was measured in m/s, step time variability was calculated as the standard deviation of step time, accuracy was calculated as the percent of subtractions problem answered correctly, and total number attempted was the total number of sutraction problems the participant attempted to answer.

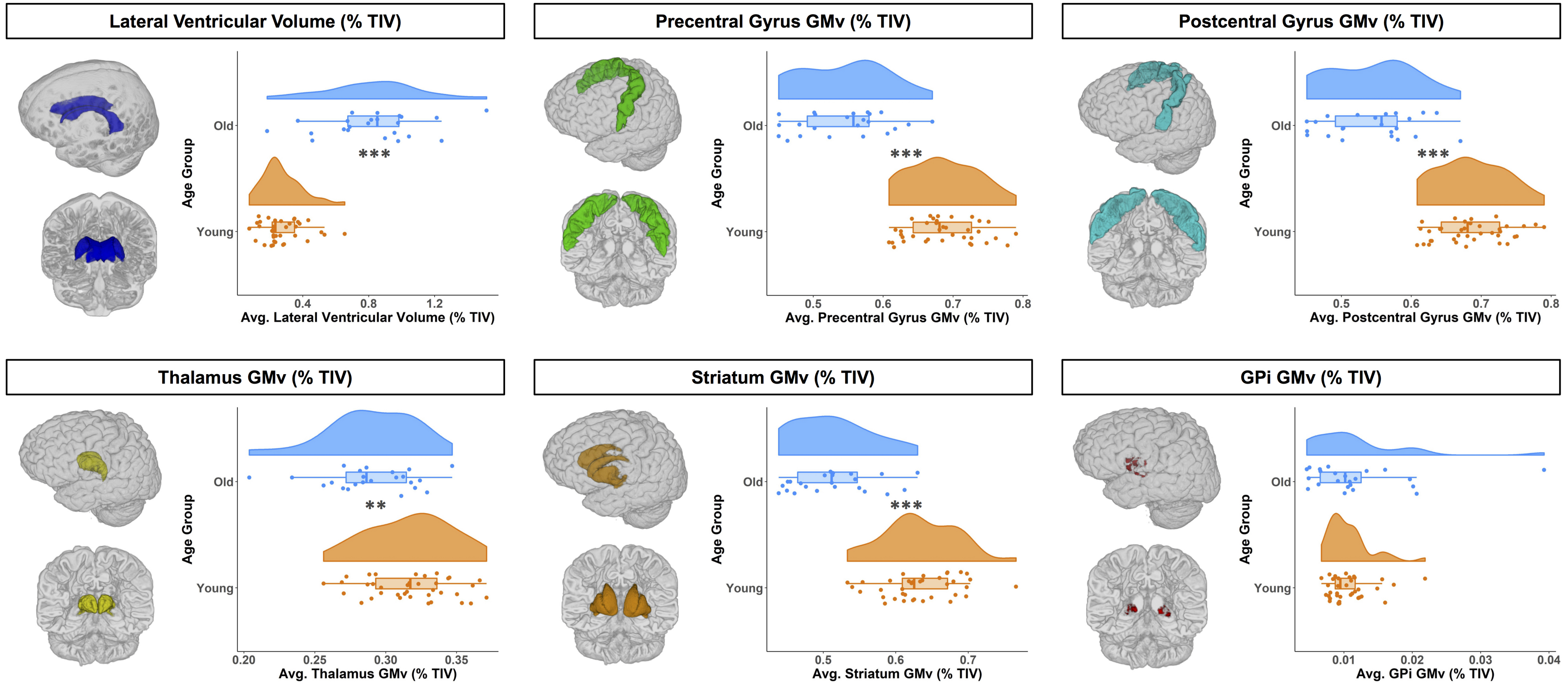

Figure S3. Age differences in ventricular volume and gray matter volume ROIs. For illustrative purposes, on the left we depict each structural ROI mask for a single exemplar subject overlaid onto that subject's native space cerebrospinal fluid (lateral ventricles) or gray matter segment (all other ROIs). On the right, we depict ROI gray matter volume (expressed as a percentage of total intracranial volume) for young (orange) and older (blue) adults. Compared with young adults, older adults had larger ventricles, as well as less gray matter volume across all ROIs except for the globus pallidus (GPi). $^{**}p_{FDR-corr} < 0.01$, $^{***}p_{FDR-corr} < 0.001$.

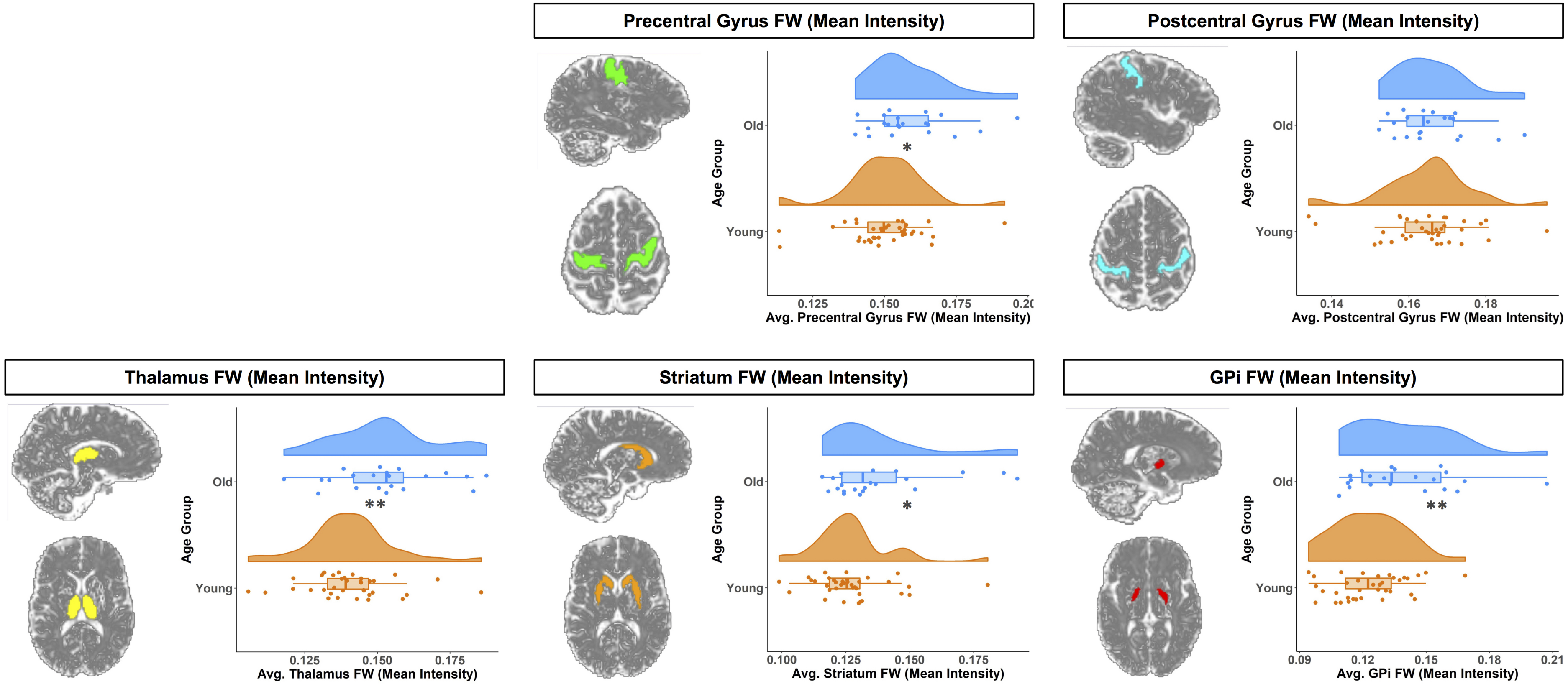

Figure S4. Age differences in FW ROIs. For illustrative purposes, on the left we depict each structural ROI mask for a single exemplar subject overlaid onto that subject's native space whole brain FW map. On the right, we depict ROI FW values (expressed as mean intensity) for young (orange) and older (blue) adults. Compared with young adults, older adults had higher FW values across all ROIs except for the postcentral gyrus. *$p_{FDR-corr} < 0.05$, **$p_{FDR-corr} < 0.01$.

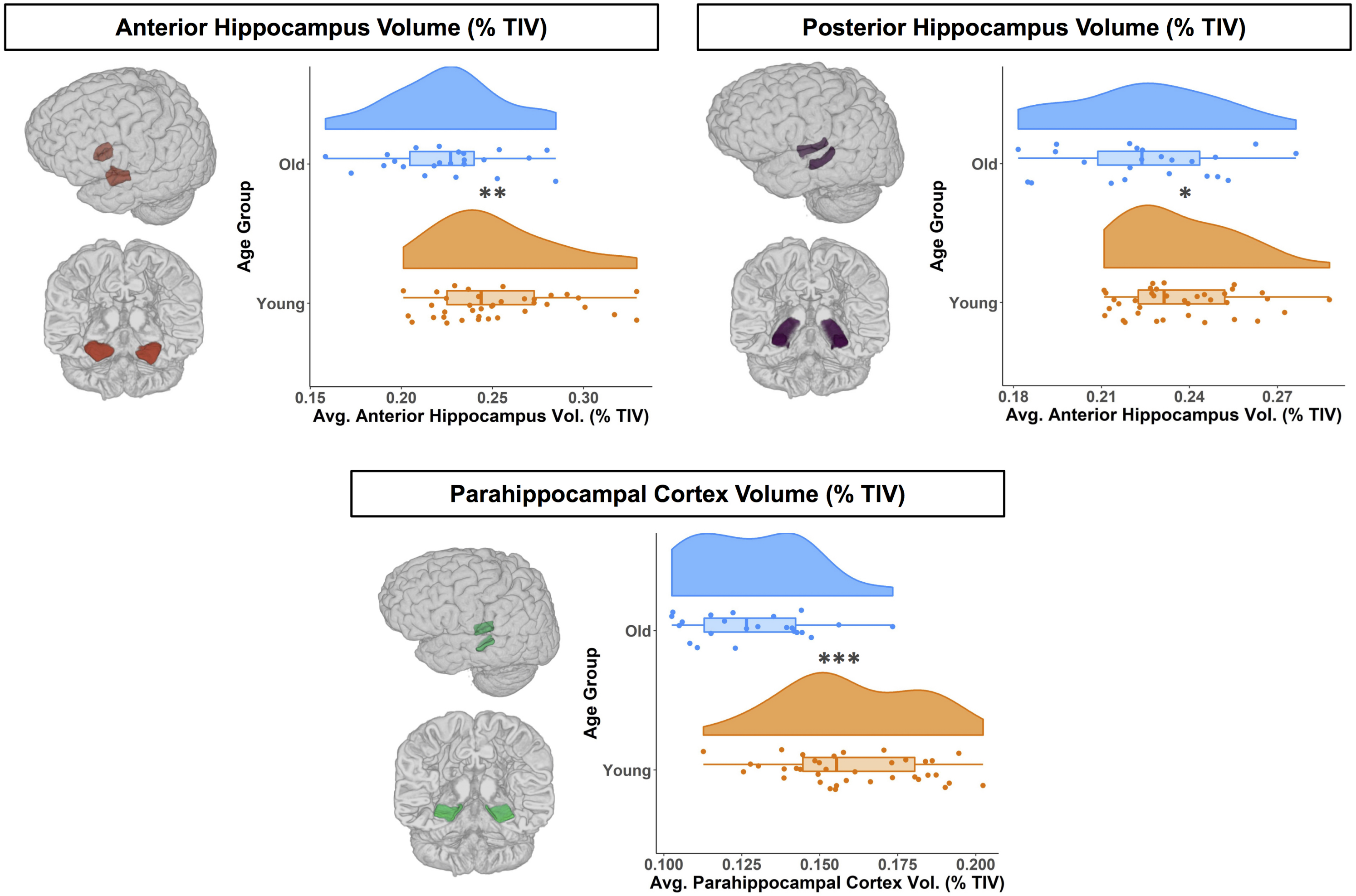

Figure S5. Age differences in hippocampal ROIs. For illustrative purposes, on the left we depict each hippocampal ROI mask for a single exemplar subject overlaid onto that subject's native space gray matter segment. On the right, we depict volume values (expressed as a percentage of total intracranial volume) for young (orange) and older (blue) adults. Compared with young adults, older adults had lower volume across all hippocampal ROIs. *$p_{FDR-corr} < 0.05$, **$p_{FDR-corr} < 0.01$, ***$p_{FDR-corr} < 0.001$.

Table S1. Age differences in structural ROIs

Mean (SD)		Predictors	Estimates (SE)	CI	t	FDR Corr. *p*	R^2
Ventricular volume (% TIV)							
Lateral ventricle		*(Intercept)*	0.40 (0.05)	0.30-0.50	7.97		
Young: 0.40 (0.18)	Old: 1.20 (0.44)	Age group *(Old)*	0.80 (0.08)	0.63-0.96	9.80	< 0.001***	
		Sex *(Male)*	-0.02 (0.04)	-0.10-0.06	-0.52	0.649	
							0.63
GM volume (% TIV)							
Precentral gyrus		*(Intercept)*	0.86 (0.01)	0.84-0.88	89.77		
Young: 0.86 (0.06)	Old: 0.64 (0.05)	Age group *(Old)*	-0.22 (0.02)	-0.25-(-0.19)	-14.07	< 0.001***	
		Sex *(Male)*	0.004 (0.01)	-0.01-0.02	0.66	0.649	
							0.78
Postcentral gyrus		*(Intercept)*	0.69 (0.01)	0.67-0.70	76.71		
Young: 0.69 (0.05)	Old: 0.54 (0.06)	Age group *(Old)*	-0.14 (0.01)	-0.17-(-0.11)	-9.93	< 0.001***	
		Sex *(Male)*	0.01 (0.01)	0.00-0.03	1.66	0.159	
							0.64
Thalamus		*(Intercept)*	0.32 (0.005)	0.31-0.33	65.70		
Young: 0.32 (0.03)	Old: 0.29 (0.03)	Age group *(Old)*	-0.03 (0.01)	-0.04-(-0.01)	-3.59	0.002**	
		Sex *(Male)*	0.01 (0.004)	0.00-0.02	2.60	0.042*	
							0.26
Striatum		*(Intercept)*	0.63 (0.01)	0.62-0.65	75.10		
Young: 0.63 (0.05)	Old: 0.51 (0.06)	Age group *(Old)*	-0.12 (0.01)	-0.1-(-0.09)	-8.86	< 0.001***	
		Sex *(Male)*	0.01 (0.01)	0.00-0.03	2.17	0.080	
							0.59
Globus pallidus		*(Intercept)*	0.01 (0.001)	0.01-0.01	12.03		
Young: 0.01 (0.003)	Old: 0.01 (0.01)	Age group *(Old)*	0.001 (0.001)	0.00-0.00	0.69	0.530	
		Sex *(Male)*	0.0001 (0.001)	0.00-0.00	0.17	0.866	
							0.01
FW (mean intensity)							
Precentral gyrus		*(Intercept)*	0.15 (0.002)	0.15-0.15	69.76		
Young: 0.15 (0.01)	Old: 0.16 (0.01)	Age group *(Old)*	0.01 (0.004)	0.00-0.02	2.41	0.028*	
		Sex *(Male)*	0.01 (0.002)	0.00-0.01	3.46	0.013*	
							0.25
Postcentral gyrus		*(Intercept)*	0.16 (0.002)	0.16-0.17	98.93		
Young: 0.16 (0.01)	Old: 0.17 (0.01)	Age group *(Old)*	0.001 (0.003)	0.00-0.01	0.43	0.667	
		Sex *(Male)*	0.004 (0.001)	0.00-0.01	3.20	0.013*	
							0.16

Table S1. Continued

Mean (SD)		Predictors	Estimates (SE)	CI	t	FDR Corr. *p*	R^2
FW (continued)							
Thalamus		*(Intercept)*	0.14 (0.003)	0.13-0.15	55.65		
Young: 0.14 (0.01)	Old: 0.15 (0.02)	Age group *(Old)*	0.01 (0.004)	0.00-0.02	3.00	0.007**	
		Sex *(Male)*	0.01 (0.002)	0.00-0.01	3.12	0.013*	
							0.26
Striatum		*(Intercept)*	0.13 (0.003)	0.12-0.13	44.48		
Young: 0.13 (0.01)	Old: 0.14 (0.02)	Age group *(Old)*	0.01 (0.005)	0.00-0.02	2.33	0.030*	
		Sex *(Male)*	0.004 (0.002)	-0.00-0.01	1.98	0.101	
							0.15
Globus pallidus		*(Intercept)*	0.12 (0.003)	0.12-0.13	36.41		
Young: 0.12 (0.02)	Old: 0.14 (0.02)	Age group *(Old)*	0.02 (0.01)	0.00-0.03	2.90	0.008**	
		Sex *(Male)*	0.001 (0.003)	-0.00-0.01	0.55	0.649	
							0.14
Hippocampal Volume (% TIV)							
Ant. hippocampus		*(Intercept)*	0.25 (0.01)	0.24-0.26	47.64		
Young: 0.25 (0.03)	Old: 0.22 (0.03)	Age group *(Old)*	-0.03 (0.01)	-0.04-(0.01)	-3.18	0.005**	
		Sex *(Male)*	-0.01 (0.004)	-0.02-0.00	-1.94	0.101	
							0.20
Post. hippocampus		*(Intercept)*	0.24 (0.004)	0.23-0.24	65.85		
Young: 0.24 (0.02)	Old: 0.22 (0.03)	Age group *(Old)*	-0.01 (0.01)	-0.02-0.00	-2.15	0.042*	
		Sex *(Male)*	0.003 (0.003)	-0.00-0.01	1.14	0.365	
							0.09
Parahippo. cortex		*(Intercept)*	0.6 (0.003)	0.15-0.17	48.17		
Young: 0.16 (0.02)	Old: 0.13 (0.02)	Age group *(Old)*	-0.03 (0.01)	-0.04-(-0.02)	-5.97	$<$ 0.001***	
		Sex *(Male)*	0.01 (0.003)	0.00-0.01	2.25	0.080	
							0.42

Note: On the left, we report the mean (standard deviation) for the young and older age groups. On the right, we report the results of a linear model testing for age group differences in each ROI, controlling for sex. *P* values for the age group and sex predictors were FDR-corrected (Benjamini and Hochberg, 1995). SE = standard error; CI = 95% confidence interval; TIV = total intracranial volume; Ant = anterior; Post = posterior; Parahippo = parahippocampal. *$p_{FDR-corr} < 0.05$, **$p_{FDR-corr} < 0.01$, ***$p_{FDR-corr} < 0.001$.

Table S2. Regions of age difference in the relationship of structural ROIs with the DTcost of step time variability

	Predictors	Estimates (SE)	t	FDR corr. *p*
Ventricular volume (% TIV)				
Lateral ventricle	DTcost variability*age group	0.003 (0.001)	1.91	0.433
GM volume (% TIV)				
Precentral gyrus	DTcost variability*age group	-0.0004 (0.0003)	-1.40	0.553
Postcentral gyrus	DTcost variability*age group	-0.0001 (0.0003)	-0.32	0.886
Thalamus	DTcost variability*age group	-0.0002 (0.0001)	-1.61	0.524
Striatum	DTcost variability*age group	0.0001 (0.0002)	0.43	0.886
Globus pallidus	DTcost variability*age group	0.00001 (0.00003)	0.31	0.886
FW (mean intensity)				
Precentral gyrus	DTcost variability*age group	0.00001 (0.0001)	0.18	0.907
Postcentral gyrus	DTcost variability*age group	-0.00002 (0.00005)	-0.51	0.886
Thalamus	DTcost variability*age group	-0.00001 (0.0001)	-0.12	0.907
Striatum	DTcost variability*age group	0.0001 (0.0001)	1.31	0.553
Globus pallidus	DTcost variability*age group	-0.00004 (0.0001)	-0.41	0.886
Hippocampal volume (% TIV)				
Ant. hippocampus	DTcost variability*age group	-0.0001 (0.0001)	-0.97	0.780
Post. hippocampus	DTcost variability*age group	-0.0001 (0.0001)	-0.86	0.786
Parahippo. cortex	DTcost variability*age group	-0.0002 (0.0001)	-2.11	0.433

Note: Here we report the results of linear models testing for age differences in the DTcost of step time variability, controlling for sex. For conciseness, we report only the estimates (standard error, SE), t, and *p* values for the statistical test of interest: the interaction of age group with the DTcost of step time variability. *P* values for the interaction term were FDR-corrected (Benjamini and Hochberg, 1995). TIV = total intracranial volume; Ant = anterior; Post = posterior; Parahippo = parahippocampal. *$p_{FDR-corr} < 0.05$.

SUPPLEMENTAL REFERENCES

Andersson, J. L., Graham, M. S., Drobnjak, I., Zhang, H., and Campbell, J. (2018). Susceptibility-induced distortion that varies due to motion: Correction in diffusion MR without acquiring additional data. *NeuroImage* 171, 277–295. doi:10.1016/j.neuroimage.2017.12.0401

Andersson, J. L., Graham, M. S., Drobnjak, I., Zhang, H., Filippini, N., and Bastiani, M. (2017). Towards a comprehensive framework for movement and distortion correction of diffusion MR images: Within volume movement. *NeuroImage* 152, 450–466. doi:10.1016/j.neuroimage.2017.02.085

Andersson, J. L., Graham, M. S., Zsoldos, E., and Sotiropoulos, S. N. (2016). Incorporating outlier detection and replacement into a non-parametric framework for movement and distortion correction of diffusion MR images. *NeuroImage* 141, 556–572. doi:10.1016/j.neuroimage.2016.06.058

Andersson, J. L., Jenkinson, M., Smith, S., and Andersson, J. (2007a). Non-linear optimisation. FMRIB technical report tr07ja1. *FMRIB Analysis Group of the University of Oxford*

Andersson, J. L., Jenkinson, M., Smith, S., et al. (2007b). Non-linear registration, aka spatial normalisation FMRIB technical report tr07ja2. *FMRIB Analysis Group of the University of Oxford*

Andersson, J. L., Skare, S., and Ashburner, J. (2003). How to correct susceptibility distortions in spin-echo echo-planar images: application to diffusion tensor imaging. *NeuroImage* 20, 870–888. doi:10.1016/S1053-8119(03)00336-7

Andersson, J. L. and Sotiropoulos, S. N. (2016). An integrated approach to correction for off-resonance effects and subject movement in diffusion MR imaging. *NeuroImage* 125, 1063–1078. doi:10.1016/j.neuroimage.2015.10.019

Ashburner, J., Barnes, G., Chen, C.-C., Daunizeau, J., Flandin, G., Friston, K., et al. (2014). SPM12 manual. *Wellcome Trust Centre for Neuroimaging, London, UK* 2464

Avants, B. B., Tustison, N. J., Song, G., Cook, P. A., Klein, A., and Gee, J. C. (2011). A reproducible evaluation of ANTs similarity metric performance in brain image registration. *NeuroImage* 54, 2033–2044. doi:10.1016/j.neuroimage.2010.09.025

Avants, B. B., Yushkevich, P., Pluta, J., Minkoff, D., Korczykowski, M., Detre, J., et al. (2010). The optimal template effect in hippocampus studies of diseased populations. *NeuroImage* 49, 2457–2466. doi:10.1016/j.neuroimage.2009.09.062

Benjamini, Y. and Hochberg, Y. (1995). Controlling the false discovery rate: a practical and powerful approach to multiple testing. *J. R. Stat. Soc. Series B Stat. Methodol.* 57, 289–300. doi:10.1111/j.2517-6161.1995.tb02031.x

Carson, N., Leach, L., and Murphy, K. J. (2018). A re-examination of montreal cognitive assessment (MoCA) cutoff scores. *Int. J. Geriatr. Psychiatry* 33, 379–388. doi:10.1002/gps.4756

Dahnke, R., Yotter, R. A., and Gaser, C. (2013). Cortical thickness and central surface estimation. *NeuroImage* 65, 336–348. doi:10.1016/j.neuroimage.2012.09.050

de Jager, C. A., Budge, M. M., and Clarke, R. (2003). Utility of TICS-M for the assessment of cognitive function in older adults. *Int. J. Geriatr. Psychiatry* 18, 318–324. doi:10.1002/gps.830

Diedrichsen, J. (2006). A spatially unbiased atlas template of the human cerebellum. *NeuroImage* 33, 127–138. doi:10.1016/j.neuroimage.2006.05.056

Diedrichsen, J., Balsters, J. H., Flavell, J., Cussans, E., and Ramnani, N. (2009). A probabilistic MR atlas of the human cerebellum. *NeuroImage* 46, 39–46. doi:10.1016/j.neuroimage.2009.01.045

Gaser, C., Dahnke, R., et al. (2016). CAT-a computational anatomy toolbox for the analysis of structural MRI data. *Hum. Brain Mapp.* 2016, 336–348

Gaser, C. and Kurth, F. (2017). *Manual computational anatomy toolbox-CAT12* (Structural Brain Mapping Group at the Departments of Psychiatry and Neurology, University of Jena)

Hupfeld, K., McGregor, H., Koppelmans, V., Beltran, N., Kofman, I., De Dios, Y., et al. (2021). Brain and behavioral evidence for reweighting of vestibular inputs with long-duration spaceflight. *Cereb. Cortex* , bhab239doi:10.1093/cercor/bhab239

Leemans, A., Jeurissen, B., Sijbers, J., and Jones, D. (2009). ExploreDTI: a graphical toolbox for processing, analyzing, and visualizing diffusion MR data. *Proc. Int. Soc. Magn. Reson. Med. Sci.* 17

Luders, E., Thompson, P. M., Narr, K., Toga, A. W., Jancke, L., and Gaser, C. (2006). A curvature-based approach to estimate local gyrification on the cortical surface. *NeuroImage* 29, 1224–1230. doi:10.1016/j.neuroimage.2005.08.049

Nasreddine, Z. S., Phillips, N. A., Bédirian, V., Charbonneau, S., Whitehead, V., Collin, I., et al. (2005). The montreal cognitive assessment, MoCA: a brief screening tool for mild cognitive impairment. *J. Am. Geriatr. Soc .* 53, 695–699. doi:10.1111/j.1532-5415.2005.53221.x

Pasternak, O., Sochen, N., Gur, Y., Intrator, N., and Assaf, Y. (2009). Free water elimination and mapping from diffusion MRI. *Magn. Reson. Imaging* 62, 717–730. doi:10.1002/mrm.22055

Romero, J. E., Coupé, P., Giraud, R., Ta, V.-T., Fonov, V., Park, M. T. M., et al. (2017). CERES: a new cerebellum lobule segmentation method. *NeuroImage* 147, 916–924. doi:10.1016/j.neuroimage.2016.11.003

Rueckert, D., Sonoda, L. I., Hayes, C., Hill, D. L., Leach, M. O., and Hawkes, D. J. (1999). Nonrigid registration using free-form deformations: application to breast MR images. *IEEE T. Med. Imaging* 18, 712–721. doi:10.1109/42.796284

Salazar, A. P., Hupfeld, K. E., Lee, J. K., Banker, L. A., Tays, G. D., Beltran, N. E., et al. (2021). Visuomotor adaptation brain changes during a spaceflight analog with elevated carbon dioxide (co_2): A pilot study. *Front. in Neural Circuits* 15, 51. doi:10.3389/fncir.2021.659557

Salazar, A. P., Hupfeld, K. E., Lee, J. K., Beltran, N. E., Kofman, I. S., De Dios, Y. E., et al. (2020). Neural working memory changes during a spaceflight analog with elevated carbon dioxide: a pilot study. *Frontiers in Systems Neuroscience* 14, 48. doi:10.3389/fnsys.2020.00048

Smith, S. M., Jenkinson, M., Johansen-Berg, H., Rueckert, D., Nichols, T. E., Mackay, C. E., et al. (2006). Tract-based spatial statistics: voxelwise analysis of multi-subject diffusion data. *NeuroImage* 31, 1487–1505. doi:10.1016/j.neuroimage.2006.02.024

Tullo, S., Devenyi, G. A., Patel, R., Park, M. T. M., Collins, D. L., and Chakravarty, M. M. (2018). Warping an atlas derived from serial histology to 5 high-resolution MRIs. *Sci. Data* 5, 1–10. doi:10.1038/sdata.2018.107

Vos, S. B., Tax, C. M., Luijten, P. R., Ourselin, S., Leemans, A., and Froeling, M. (2017). The importance of correcting for signal drift in diffusion MRI. *Magn. Reson. Imaging* 77, 285–299. doi:10.1002/mrm.26124

Yotter, R. A., Dahnke, R., Thompson, P. M., and Gaser, C. (2011a). Topological correction of brain surface meshes using spherical harmonics. *Hum. Brain Mapp.* 32, 1109–1124. doi:10.1002/hbm.21095

Yotter, R. A., Nenadic, I., Ziegler, G., Thompson, P. M., and Gaser, C. (2011b). Local cortical surface complexity maps from spherical harmonic reconstructions. *NeuroImage* 56, 961–973. doi:10.1016/j.neuroimage.2011.02.007

Yun, H. J., Im, K., Yang, J.-J., Yoon, U., and Lee, J.-M. (2013). Automated sulcal depth measurement on cortical surface reflecting geometrical properties of sulci. *PloS One* 8, e55977. doi:10.1371/journal.pone.0055977

Yushkevich, P. A., Piven, J., Hazlett, H. C., Smith, R. G., Ho, S., Gee, J. C., et al. (2006). User-guided 3d active contour segmentation of anatomical structures: significantly improved efficiency and reliability. *NeuroImage* 31, 1116–1128. doi:10.1016/j.neuroimage.2006.01.015

Yushkevich, P. A., Pluta, J. B., Wang, H., Xie, L., Ding, S.-L., Gertje, E. C., et al. (2015). Automated volumetry and regional thickness analysis of hippocampal subfields and medial temporal cortical structures in mild cognitive impairment. *Hum. Brain Mapp.* 36, 258–287. doi:10.1002/hbm.22627
